# Fabrication and Application of Halloysite Nanotube-Embedded Photocatalytic Nanofibers with Antibacterial Properties

**DOI:** 10.1021/acsomega.2c06880

**Published:** 2022-12-26

**Authors:** Elifnur Gezmis-Yavuz, Tulay Ergon-Can, C. Elif Cansoy, Derya Y. Koseoglu-Imer

**Affiliations:** † Department of Environmental Engineering, Istanbul Technical University, Maslak Campus, Istanbul 34469, Turkey; ‡ Department of Maritime Transportation Management Engineering, Piri Reis University, Istanbul 34940, Turkey

## Abstract

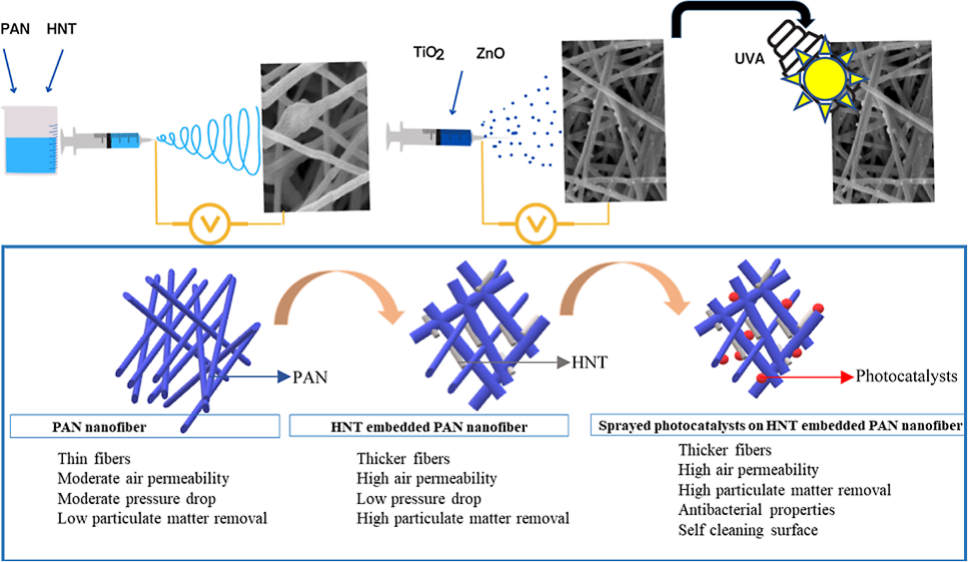

With decreasing indoor air quality, increased time spent at indoors, and especially with the COVID-19 pandemic, the development of new materials for bacteria and viruses has become even more important. Less material consumption due to the electrospinning process, the easy availability/affordability of the halloysite nanotube (HNT), and the antibacterial effect of both TiO_2_ and ZnO nanoparticles make the study even more interesting. HNTs have attracted research attention in recent years due to their low cost, high mechanical strength, natural and environmentally friendly structure, and non-toxicity to human health and ecosystem. In this study, HNT-embedded composite nanofiber filters were fabricated as filter materials using the electrospinning method. Photocatalysts (TiO_2_ and ZnO) were incorporated into the composite nanofibers by the electrospraying method. The results showed that the combination of both HNT/TiO_2_ and HNT/ZnO additives was successfully integrated into the filter structure. The effect of embedding the HNT and spraying photocatalysts enables the fabrication of composite filters with lower pressure drop, high filtration efficiency, improved mechanical properties, and high antibacterial properties against *Escherichia coli*, making the nanofibers suitable and promising for face masks and air filter materials.

## 1 Introduction

Airborne diseases caused by bacteria and viruses and their deadly effects on human health have led to antibacterial materials occupying an important place in public health. The main pollutants such as bioaerosols, particulate matter (PM), volatile organic compounds (VOCs), and CO_2_ in indoor air have become the subject of numerous studies.^1^ In recent years, various filter structures have been used to remove these pollutants, and nanofiber filters have also shown promise due to their unique properties such as a very high surface-to-volume ratio, high permeability, low basis weight, and nanoporous structure.^2−4^ Electrospun nanofibers are used to remove VOCs, chemical and biological pollutants, and toxic substances from indoor air.^4−9^ Depending on the removal of target pollutants in indoor air, various polymers and nanoadditives can be used in the production of electrospun nanofiber filters.

Many natural and synthetic polymers such as polyacrylonitrile (PAN), polyamide (PA), polyvinyl alcohol (PVA), polyethersulfone, polyvinyl chloride, polycarbonate (PC), polyurethane (PU), polyvinylidene fluoride, cellulose (CA), chitosan (CS), and protein are preferred as bare and composite materials in the indoor air filter fabrication with the electrospinning process.^2,5,9−16^ Among these polymers, especially, PAN stands out and it is a semicrystalline polymer and has high thermal stability, resistance to most solvents, widely used commercially, easily spinnable, and frequently used in air filtration.^17−19^ PAN polymer has a good compatibility with many nanoadditives.^4,20−24^ Some metal oxides have been preferred in the fabrication of PAN nanofibers to achieve the photocatalytic properties and antibacterial capabilities of filters.^25^ The wide-use materials that impart photocatalytic properties to nanofibers are ZnS, CdS, Fe_2_O_3_, WO_3_, and TiO_2_.^26^ Due to their low cost and high stability, TiO_2_ and ZnO are considered as the most preferred photocatalysts, and successful filters have been obtained for the indoor air filter.^11,27,28^

In recent years, nanoclays have been integrated to filter materials due to their high aspect ratio, ease of application, low cost, good dispersion and non-toxicity properties.^29,30^ HNT is a member of the clay family that attracts attention because of its natural availability and hollow tubular structure in nature and consists mainly of aluminosilicate with the chemical formula Al_2_Si_2_O_5_(OH)_4_·*n*H_2_O.^31^ Halloysite nanotubes (HNT) have a bilayer crystallographic structure of tetrahedral silicate and octahedral aluminum hydroxide layers separated by water molecules.^32^ HNT has begun to attract research attention as a nanoadditive in recent years. HNT nanoadditives are preferred in different application areas like sensor development, protective equipment, food package industry, drug delivery and filter material, dyes removal, especially in biomedical applications^33−37^ due to its low cost, high mechanical properties, environmental friendliness, and harmlessness to human health. HNTs are easier to disperse than other plated clays that exhibit exfoliation in polymer matrices. Because of the relatively few hydroxyl groups on their outer surface compared to other clay minerals, they are easy to disperse and particularly important for nanofiber structures fabricated from a polymer solution containing HNTs.^38,39^ There are many studies on the use of nanoclay in electrospinning processes with different polymers. There is a gap in the literature about the applications of nanoclays and HNT-embedded nanofibers at face masks and indoor air filtration. HNTs are among the promising materials for adsorption due to the surfaces of the nanotube cavities. Although there are studies using HNTs to remove pollutants from the aquatic environment in environmental applications, there are gaps in the literature for airborne contaminant filtration studies.^40,41^ There are studies on natural organic matter removal and photodegradation in water applications using HNT/TiO_2_ and HNT/ZnO.^42−45^ The high adsorption capacity of HNTs due to their high surface area increases the decomposition of organic pollutants for photocatalytic degradation with the use of TiO_2_.^46^ TiO_2_/HNTs are used for their ion exchange capacity due to their high specific surface area and slightly negative charge.^43^

Our research has mainly focused on the combined use of photocatalysts and HNTs for air filter materials. In this study, PAN/HNT/TiO_2_ and PAN/HNT/ZnO filters have been fabricated with high removal efficiency of fine (PM 0.3), antibacterial properties, low pressure drop values, and high air permeability were fabricated. The novelties of the study are (i) integration of photocatalysts into HNT-embedded nanofibers by electrospraying technique; (ii) fabrication of PAN/HNT nanofiber filters with high antibacterial properties under UVA light with electrosprayed TiO_2_ and ZnO additives; (iii) PAN/HNT with different photocatalysts nanofibers that have not been used before as air filter materials; and (iv) high filtration efficiency and low pressure drop values of the fabricated nanofibers.

## 2 Materials and Methods

### 2.1 Materials

PAN was purchased from Sigma-Aldrich and *N*,*N*-dimethylformamide (DMF) (99.8% purity) from Merck to prepare the polymer solutions. HNTs (20–150 nm in diameter and 100–600 nm in length) were supplied from ESAN Company in Turkey. TiO_2_ (CAS:1317-70-0), ZnO (CAS: 1314-13-2), and ethanol (purity: ≥99.9%) were purchased from Sigma-Aldrich and Merck, for electrospraying, respectively. TiO_2_ is in the form of anatase, and its particle size is in the range of 100–200 nm. The nonwoven support layer was purchased from MOGUL. All materials were used as received without any purification.

### 2.2 Fabrication of Electrospun Nanofiber Filters

First, PAN (12% wt) was dissolved in DMF and stirred for 8 h at room temperature for the preparation of bare nanofibers. HNTs were dispersed in DMF at different ratios of 1, 3, and 5 wt % by using a sonication probe (Hielscher, model UP200St) for 20 min at an amplitude of 20. Then, the PAN polymer was added to HNT dispersion and stirred for 8 h at room temperature. The electrospinning parameters are chosen as follows: needle collector distance 19 cm; applied voltage 16 kV; feed rate 0.75 mL/h, and time 1 h. The support layer, which is used to collect the nanofibers on its surface weighs 30 g/m^2^. After the electrospinning process, TiO_2_ and ZnO photocatalysts were electrosprayed onto the bare PAN and PAN/HNT composite filters. 10 wt % photocatalyst was dispersed in ethanol and sprayed at a feed rate of 5 mL/h for 30 min to prepare all filters by using Inovenso NS24 model electrospinning/electrospraying equipment. All PAN based nanofiber filters were indicated as given in Table 1.

**Table 1:** Composition of Bare and Nanocomposite PAN Nanofibers

	HNT	photocatalyst	
filter name	amount (% wt)	type	amount (% wt)
PAN			
HNT1	1		
HNT3	3		
HNT5	5		
PAN-TiO_2_		TiO_2_	10
HNT1-TiO_2_	1	TiO_2_	10
HNT3-TiO_2_	3	TiO_2_	10
HNT5-TiO_2_	5	TiO_2_	10
PAN-ZnO		ZnO	10
HNT1-ZnO	1	ZnO	10
HNT3-ZnO	3	ZnO	10
HNT5-ZnO	5	ZnO	10

### 2.3 Characterization Methods

The fiber morphology was characterized by using a scanning electron microscope (SEM, Philips, XL30SFEG). Image J software (1.52a JAVA 1.8.0_112, National Institutes of Health, USA) was used to quantify fiber diameters at 50 different points from SEM pictures. SEM–EDS was used to determine the elemental composition distributions of nano-additives. The Fourier transform infrared spectroscopy (FTIR) of the bare and composite nanofiber filters were analyzed using a PerkinElmer spectrum 100 spectrophotometer. The absorption peaks in the FTIR spectrum correspond to the frequencies created by the vibration of the bonds between the atoms of the material. Substances have a distinct spectrum, with the area after 2000 cm^–1^ being the most detailed; hence, this area is referred to as the “fingerprint”. The data obtained in the FTIR investigation represents different vibrational frequencies emanating from different chemical bonds. Thermogravimetric analysis (TGA) analysis was performed using the PerkinElmer diamond thermal analysis system. It was operated at a heating rate of 10 °C/min and a temperature range of 20–1000 °C. The mechanical strength of the samples was tested with the Instron 3345 device. The nanofiber filter samples were cut into a rectangular shape of 5 cm in length and 2 cm in width. Nanofiber filters were placed in the two jaws of the device, and a tension of 0.05 mm/s was applied to the nanofiber filters placed between the jaws to stretch the samples. The load cell was kept at 500 N at room temperature. According to the data obtained from the device, strain versus stress graphs were drawn and tensile strength values were calculated. The air permeability of the nanofiber filter was measured in accordance with the standard TS 391 EN ISO 9237 with the ProWhite AirTest II test device. Nanofiber filters were fixed with a 20 cm^2^ round head during measurement. Air permeability measurements were taken in mm/s at a pressure of 100 kPa. Three measurements were taken from each nanofilter to obtain a representative value.

The performance of water vapor passing through a filter is evaluated by the water vapor transmission value. Water vapor transmission occurs by diffusion and is proportional to the thickness of the filter. The experiment was conducted for 1 h on a heater set at 40 °C using special equipment in accordance with the ASTM E96 standard. The water vapor transmission value (g/m^2^ h) was determined by dividing the change in weight after 1 h by the filter area. The following equations are used to measure the water vapor transmission rate
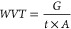
1*WVT* is for water vapor transmission rate (g/h m^2^), *G* for weight change (g), *t* for time (h), and *A* for filter test area (m^2^).^47^

### 2.4 Filtration Efficiency and Pressure Drop

The automated filter test device (TSI 8130A) was used to assess the filter efficiency and pressure drop of the nanofiber for face mask and air filters. The nanofiber filter was inserted in the lower filter holder, and the upper half of the filter was closed. The aerosol was sent from the upper part, where the particle concentration was determined at the lower part of the holder. The pressure drop was measured with a pressure transducer placed between the filter holders. Particle removal efficiency and pressure drop value were tested simultaneously. The tests were measured at 5.3 cm/s and 95 L/min flow conditions with 0.3 μm NaCl aerosol. The efficiency of a filter in the filtration process is determined by the percentage value of the pollutant released from the filter material. The amount of pollutants can be expressed as mass, particle count, or volume. The filtration efficiency was determined using eq 2, where *C* and *C*_0_ are the number of particles before and after filtration.^15,48^ Filtration efficiency is represented as
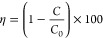
2QF was calculated using eq 3, where QF (Pa^–1^) represents the quality factor, η is the filtering efficiency (selectivity), and Δ*P* (Pa) is the pressure drop.^49^
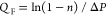
3

### 2.5 Antibacterial Activity Tests

*Escherichia coli* K12 strain was used as an indicator bacteria for the determination of antibacterial activity. *E. coli* K12 strain supplied as a freeze-dried form was grown in lysogeny broth (LB) Miller culture medium for 18 h at 37 °C under aseptic conditions. Then, the growth medium was removed by centrifugation, and the bacteria pellet was resuspended in a 0.9% saline solution to prepare stock culture. The antibacterial activity of the filters was determined using agar diffusion test and plate counting method.

Briefly, disc diffusion test was carried out by spreading 100 μL of bacterial suspension from fresh overnight grown culture of *E. coli* on the agar plate. Nanofiber filter discs (1 cm^2^) were then placed onto the overlay agar plates, and all samples were incubated at 37 °C for 24 h.

For plate counting assay, 20 μL of *E. coli* suspension was dropped onto the nanofiber filters cut into squares with an area of 1.5 cm^2^ and covered with a glass slide. To investigate the effect of exposure time to UVA light, the nanofiber samples were exposed to a UVA source for 0–30–60–90–120–240 min. As a control for this experiment, the nanofiber samples were kept under the same conditions and for the time without the influence of UVA light. Thus, the antibacterial effect of TiO_2_ and ZnO activated by the effect of UVA light could be tested. The antibacterial property of HNTs without the effect of UVA light was also determined in the same experimental systematic. After each specified time, the nanofiber samples were dipped into 10 mL of saline solution (containing 0.2% Tween 80), vortexed for 5 min, and mixed in an ultrasonic bath for 3 min. Then, the solution was serially diluted, and 100 μL of each dilution was inoculated onto an agar plate. The plates were incubated at 37 °C for 24 h, following which incubation colonies were counted for number of viable cells. The antibacterial activity of the filters was calculated according to eqs 4 and 5.
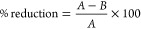
4
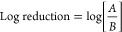
5where *A* is the number of viable bacteria (CFU) before treatment and *B* is the number of viable bacteria (CFU) after treatment.

It can be characterized as a slight decrease when the log removal values are between 0.5 and 1 of a significant decrease when the log removal values are between 1 and 3 and of a strong decrease when log removal is greater than 3.^50^

### 2.6 CLSM Method

Live and dead bacteria were visualized using a Zeiss LSM 780 (20×/0.8 NA objective) model laser scanning confocal microscope at Istanbul Medipol University. Nanofiber filters (1 cm^2^ area) were placed on the glass coverslip. Bacteria grown overnight were prepared fresh using saline. High ratios of bacterial suspension were dropped on the nanofiber filters. Then, the samples were stained with the “LIVE/DEAD BacLight Bacterial Viability Kit” (ThermoScientific, L7012) in accordance with the protocol recommended by the manufacturer in order to determine bacterial viability using the microscopic staining and imaging method. The kit contents (Propidium iodide, SYTO13) were dripped onto the samples and incubated for 10 min at room temperature; the membrane samples placed on a positively charged slide were covered with a coverslip and were scanned with a confocal laser scanning microscope (CLSM). Filters were kept under UVA exposure (9 W, Osram UVA lamp) for 1 h. As a result of staining, live (green) and dead (red) bacteria in the samples were displayed separately. Then, the images were processed to merge using ImageJ software.

## 3 Results and Discussion

### 3.1 Structural Characterization of Fabricated Nanofibers

**Figure 1:**
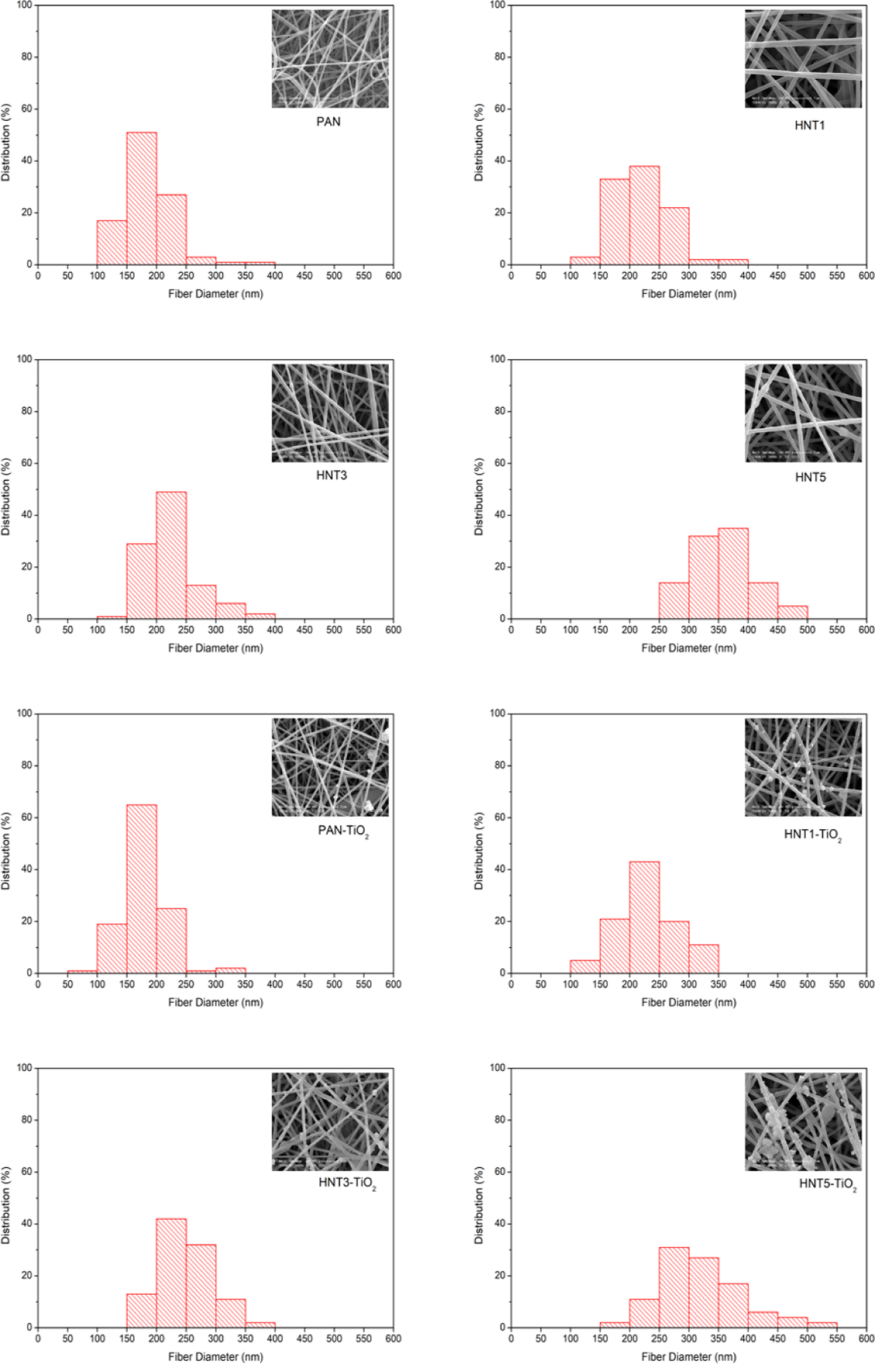

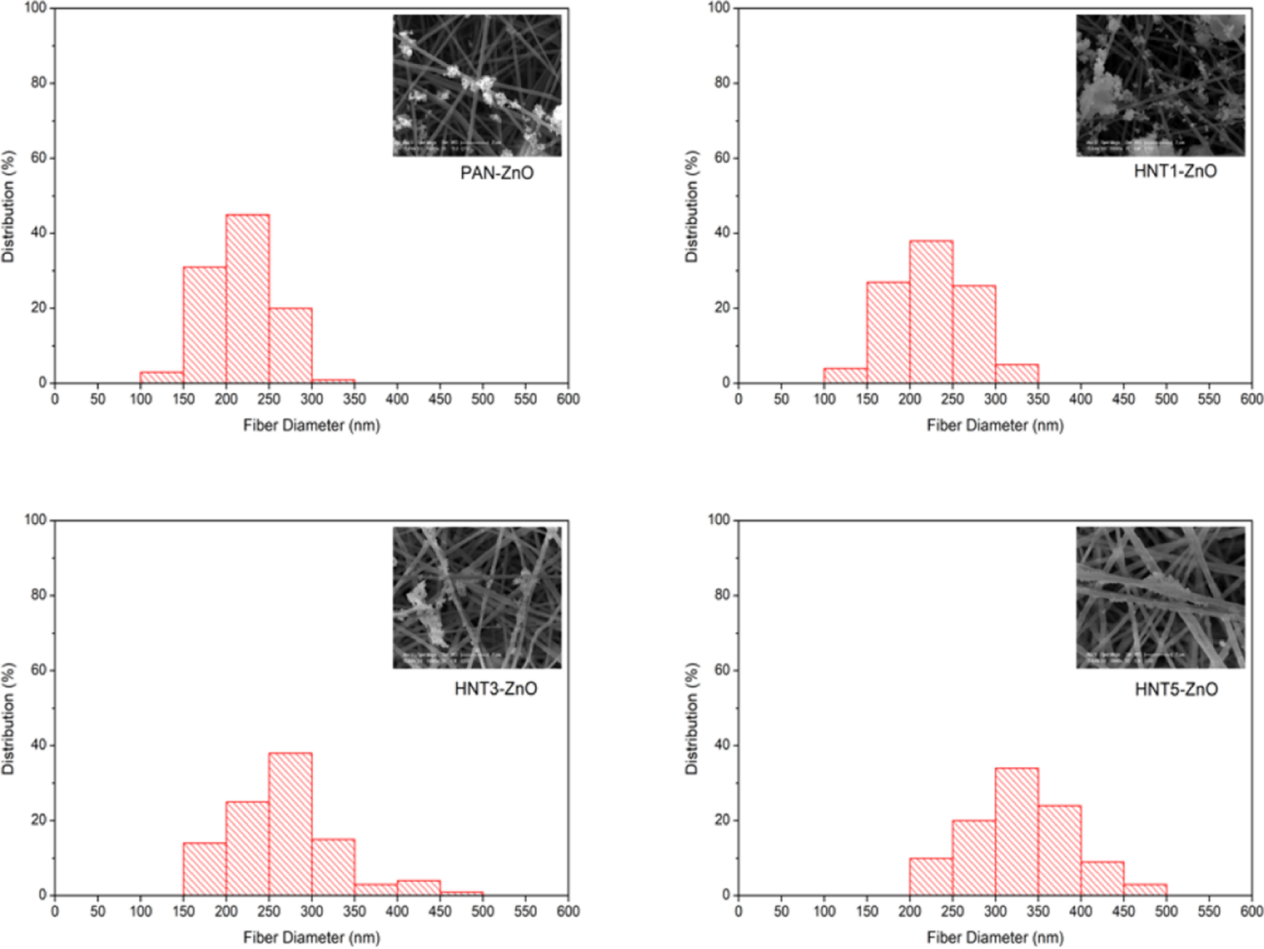
Fiber diameter distributions and SEM images of PAN composite nanofibers.

Figure 1 shows the histogram of fiber diameters and SEM images of PAN (12 wt %), HNT, HNT-TiO_2_, and HNT-ZnO-coded composite nanofiber filters. The fiber diameters were ranging between 140 and 500 nm, and an increase in fiber diameters was observed with the increase of the HNT content. While the average fiber diameter of bare PAN nanofibers was measured as 185 ± 40 nm, an addition of 5 wt % HNT caused an increase in the fiber diameters of composite nanofibers and it reached up to 360 ± 51 nm. Additionally, compared to the bare PAN/HNT nanofibers, no major change in the fiber diameter was observed when TiO_2_ and ZnO photocatalysts were electrosprayed. These findings were in concordance with the literature results. Similarly, Makaremi et al. (2015) found that the diameters of their PAN/HNT nanocomposite fibers were increased with the increase of HNT concentration from 484 to 570 nm. HNT consists of a negative charge on the external surface^52,53^ and the addition of negatively charged HNTs caused a decrease in the surface charge density and an increase in electrical conductivity and viscosity of polymer solution, so this leads to the formation of larger diameter fibers.^51^

**Figure 2:**
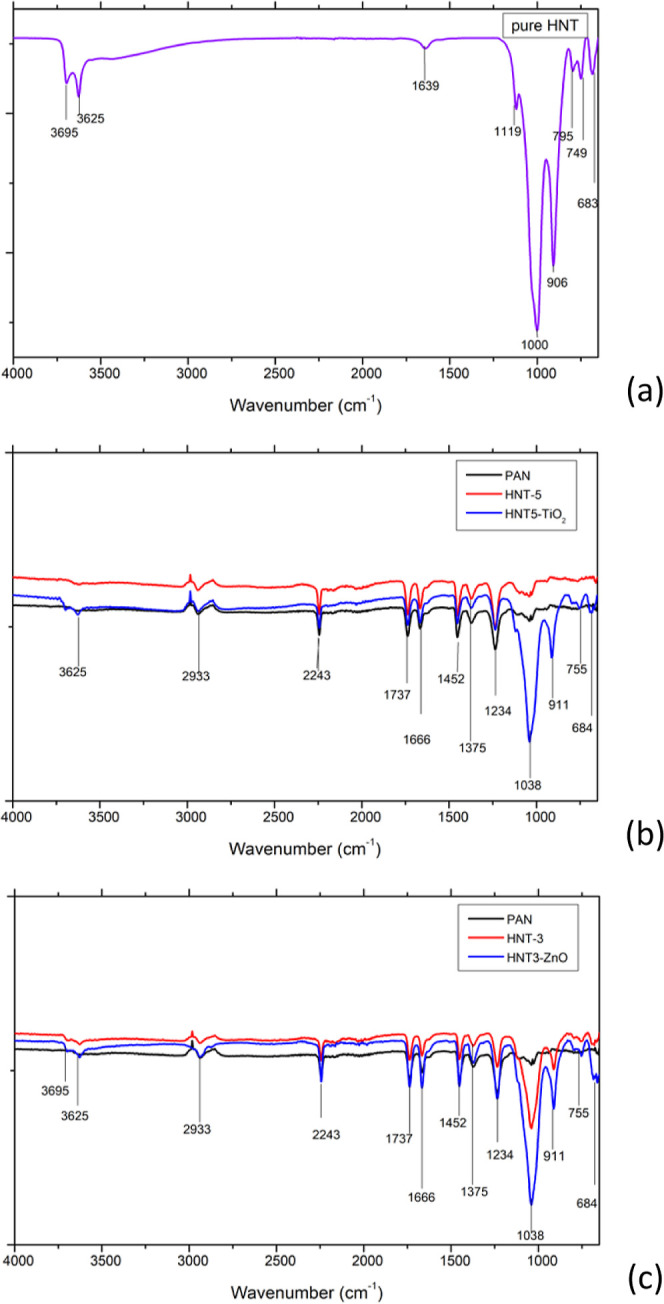
FTIR spectra for bare and composite PAN nanofibers (a) pure HNT (b) bare PAN nanofiber, HNT5 coded nanofiber, and HNT5-TiO_2_ coded nanofiber, and (c) bare PAN nanofiber, HNT3 coded nanofiber, and HNT3-ZnO coded nanofiber.

FTIR spectra of pure HNT, bare PAN nanofiber, PAN/HNT, PAN/HNT/TiO_2_, and PAN/HNT/ZnO composite nanofibers are reported in Figure 2. The 2933 and 2243 cm^–1^ vibration peaks in the FTIR spectrum indicate the nitrile (−CN) bond while the 1737 cm^–1^ peak corresponds to the C=O bond of the bare PAN nanofiber. Bare and composite nanofibers showed C–O stretching vibration at 1375 cm^–1^.^54,55^ The peak at 1666 cm^–1^ was related to the −C=C– bonds in the PAN structure. The stretching vibration of O–H on the inner surface of the HNT is related with the distinctive bands in the FTIR spectra of HNT at 3625 cm^–1^.^56,57^ The strong vibration band seen at 1000–1038 cm^–1^ can be attributed to the Si–O–Si bond originating from the HNT.^58^ Vibration bands seen at this wavenumber are not examined in the bare membrane but are measured in bare HNT powder and all of HNT composite nanofilters. The vibration band around 911 cm^–1^ can be attributed to the deformation of inner hydroxyl (O–H) groups,^59^ and 755 cm^–1^ vibration band can be observed as the stretching vibrations of Si–O.^60−62^ According to the FTIR results, it can be said that the HNT was successfully introduced within the nanofiber structure. It was observed that new peaks were formed in the 950–650 cm^–1^ band after the electrospraying of photocatalysts which indicates the presence of Ti–O–Ti.^4,63^ The addition of TiO_2_ and ZnO does not affect the wavenumber of the peaks strongly. Therefore, it can be concluded that weaker physical interactions have occurred between the photocatalysts and the polymer rather than a strong chemical bond.^64^

**Figure 3:**
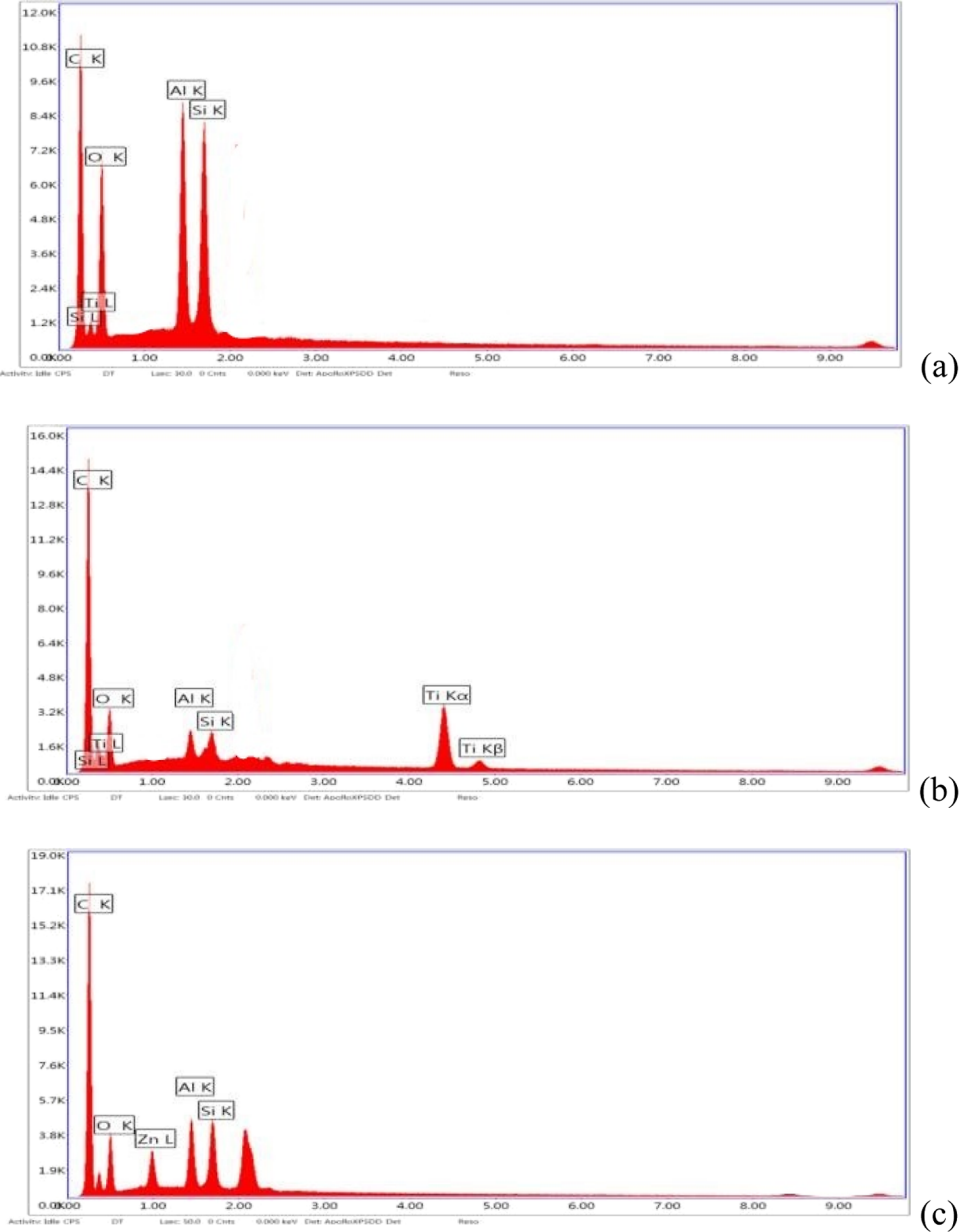
SEM–EDS result of composite nanofiber (a) HNT3, (b) HNT3-TiO_2_, and (c) HNT3-ZnO.

In addition to FTIR, SEM–EDS analysis was also performed to determine the integration of nanoadditives into the nanofiber structure. In addition to C and O present in the PAN structure, peaks of Al and Si are also evaluated as an evidence of the presence of the HNT according to the SEM-EDS results.^65−67^Figure 3a shows the SEM-EDS spectra of PAN/HNT composite nanofibers. The spectra of SEM–EDS showed that TiO_2_ and ZnO photocatalysts were successfully electrosprayed on the PAN/HNT nanofiber surface, indicating Si and Al elements for the HNT and Ti and Zn peaks for PAN/HNT/TiO_2_ (Figure 3b) and PAN/HNT/ZnO nanofibers (Figure 3c). The SEM-EDS data reveal that nanoadditives were successfully added to nanofiber filters.

**Figure 4:**
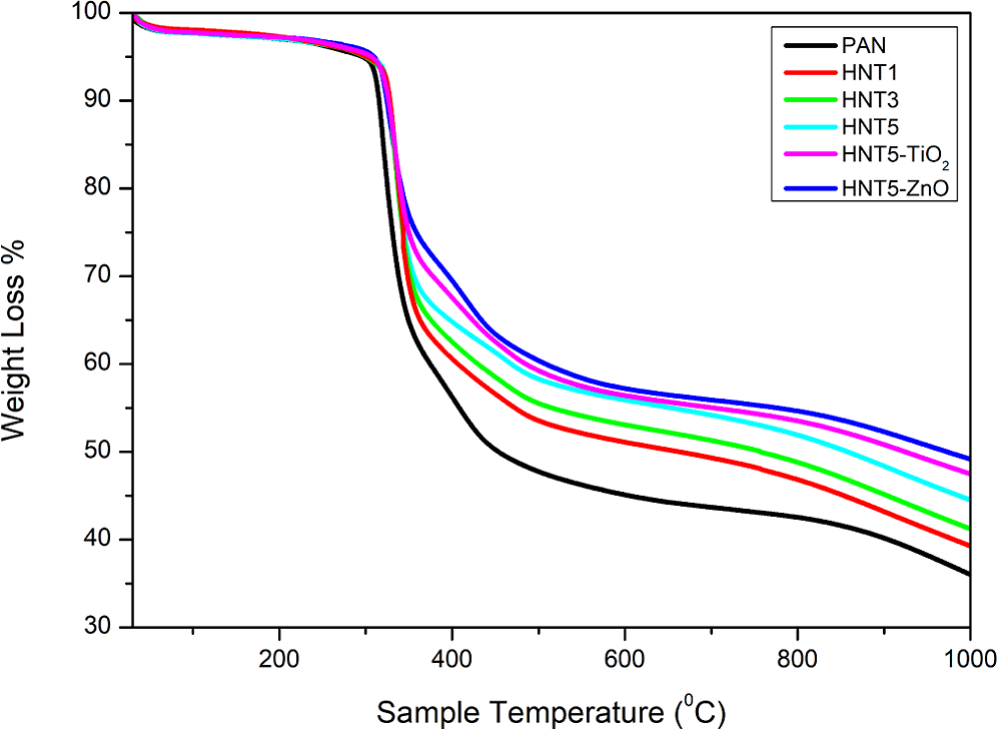
TGA curves of PAN composite nanofibers.

Thermal degradation of PAN, HNT1, HNT3, HNT5, HNT5-ZnO, and HNT5-TiO_2_-coded composite nanofibers was performed by thermogravimetric analysis as shown in Figure 4. The increased weight loss with the percentage of HNTs responded to the addition of HNT into nanofibers. The gradual loss of mass indicates the dispersion of HNTs in nanofibers. As shown in Figure 4, the HNT5-TiO_2_ and HNT5-ZnO nanofibers had a residual mass proving the presence of ZnO and TiO_2_. Furthermore, the increased residual weight responded with the percentage of HNTs to the addition of halloysite to nanofibers. This suggests that the HNT was better dispersed in the fibers. As can be seen in Figure 4, bare PAN nanofibers begins to decompose around 290 °C. Relatively higher temperatures are required for the decomposition of PAN composite fibers. It was observed that the decomposition temperature increased with the increase of HNT addition.^68^

**Figure 5:**
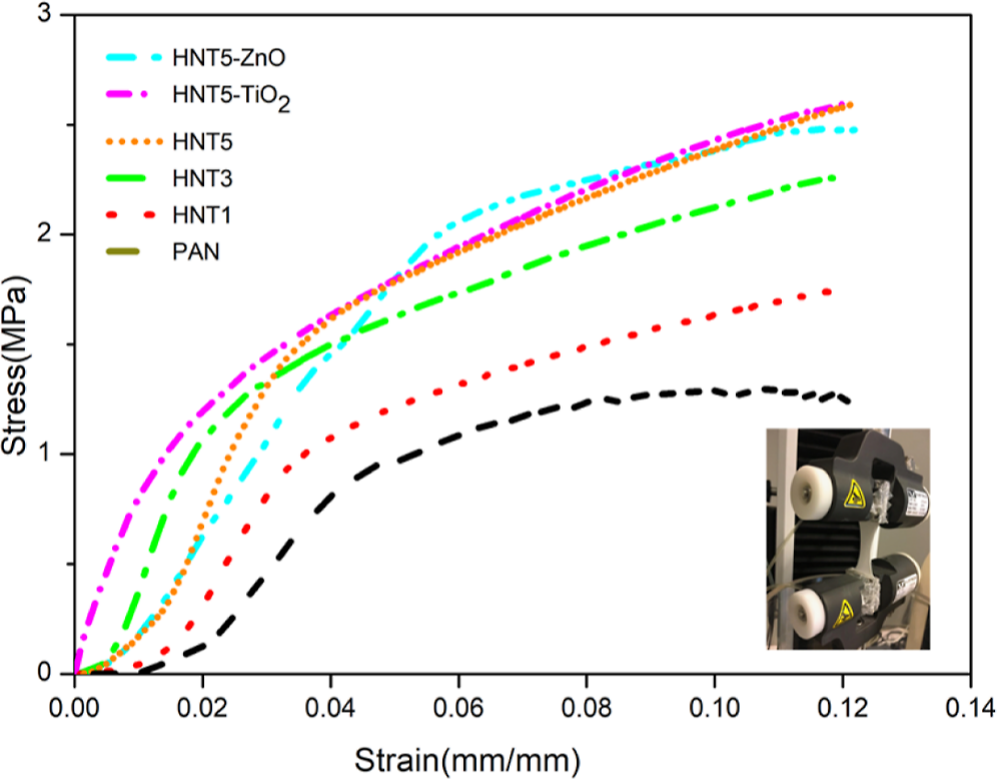
Stress–strain plots of the PAN composite electrospun nanofiber (note: photo was taken by one of the authors).

The stress–strain curve for bare PAN and HNT–PAN composite fibers is given in Figure 5. As can be seen from the graph, while the tensile strength of the bare PAN polymer was approximately 1.2 MPa, a significant increase was found in tensile strength of HNT–PAN composites with the increase of the HNT content and the values are 1.7, 2.2 and 2.6 MPa for HNT1, HNT3, and HNT5, respectively. Because the metal-oxide nanoparticles were not dispersed in polymer solution and were sprayed directly on nanofibers, the presence of metal oxide nanoparticles did not agglomerate and did not cause a decrease on mechanical strength.

### 3.2 Air Permeability and Water Vapor Transmission Rate Results

**Figure 6:**
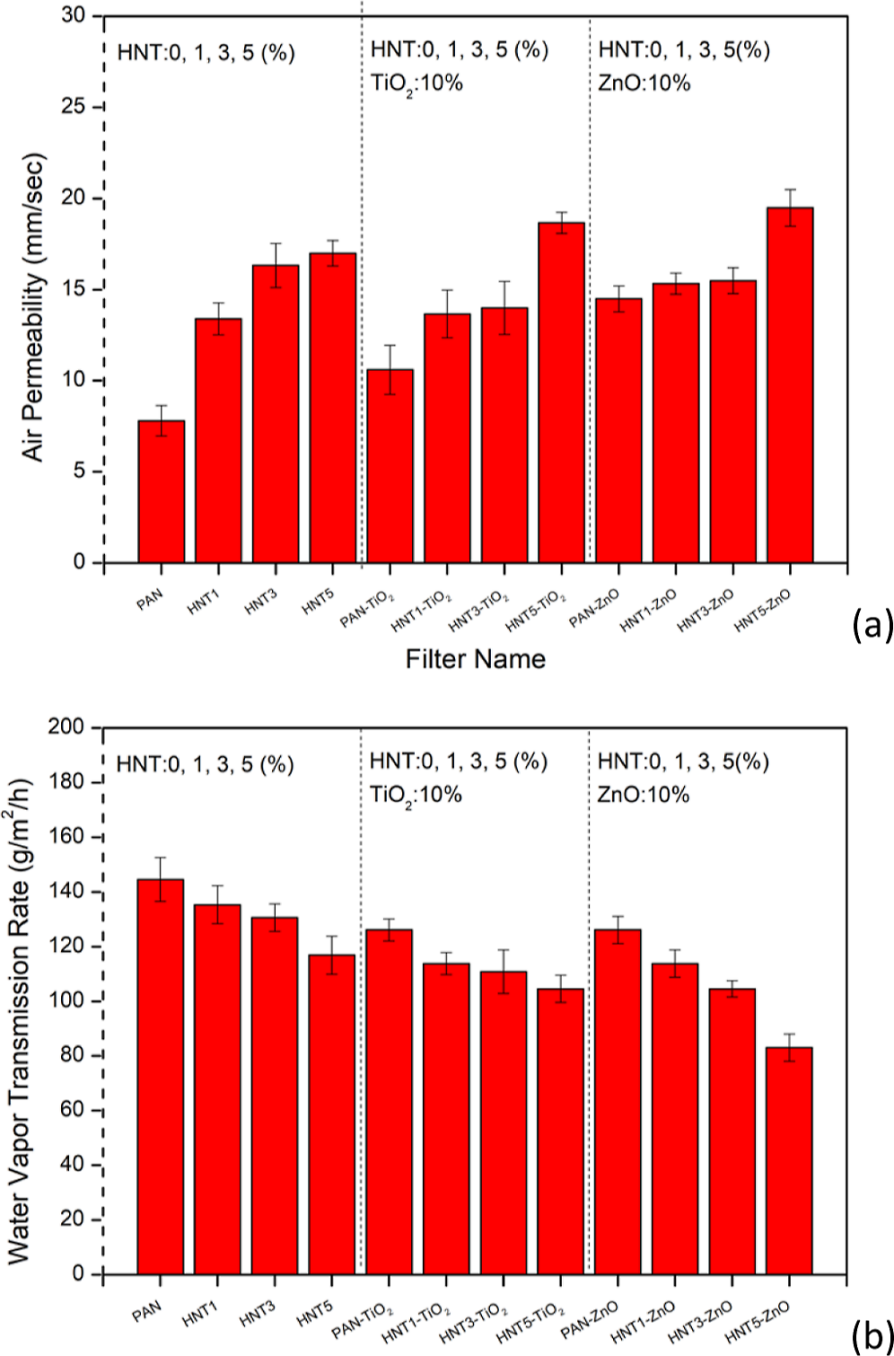
Air permeability values (a) and water vapor transmission rate of bare and composite PAN nanofiber filters (b).

Figure 6a shows the air permeability values of all filters, which were measured at a pressure of 100 kPa and obtained less than 20 mm/s. An increase was found in air permeability values of filters with the increase of the HNT amount. The air permeability of nanofibers is affected by general characteristics such as fiber diameter, membrane thickness, membrane wettability, and porosity.^21,69^ In our study, as described in Section 3.1, both the fiber diameter and air permeability values increased with the addition of the HNT. With the decrease of fiber diameter, denser fiber structures are obtained. In case of smaller fiber diameters, fiber structures may overlap and form a denser structure, and this may tend to reduce air permeability values. As the fiber diameter increases as a function of content, the number of fiber diameters per unit area decreases and the gaps between fibers increase. The air permeability value of the bare PAN (12 wt %) nanofiber filter was measured as 8 mm/s, while this value increased up to 13, 16, and 19 mm/s with the addition of HNT of 1, 3, and 5 wt %, respectively. As can be seen from Figure 6a, these values were expected to be increased with the increase of the fiber diameter. Bansal and Purwar (2021) fabricated ZnO-montmorillonite PAN nanofiber filters for PM filtration. When the amount of montmorillonite additive was increased from 0 to 1% in the prepared nanofiber filters with a concentration of 7 wt % PAN, it was reported that the values of air permeability of nanofibers increased from 5.5 to 6.6 mm/s, respectively. Wang et al. (2014) fabricated PAN/PU composite nanofibers that were fluorinated polyurethane modified to retain fine particles. The fiber diameters of PAN/PU composite nanofibers were measured to in the range of 304–558 nm as average. When compared to bare PAN nanofiber filters, the increase in the fiber diameter improved air permeability values due to increase in the pore size of composite membranes.^70^ According to our findings and as well as the literature results, it can be concluded that air permeability increases with the increase of the fiber diameter. As a result of these findings, it is well understood that the HNT is uniformly integrated within the nanofibers and the presence of HNTs improved the air permeability values of the nanofibers which can also be thought to be particularly beneficial in terms of air filtration efficiency.

As can be seen from Figure 6b, the water vapor transmission (WVT) values of all nanofibers decreased with the increase of the HNT content. HNT has a unique structure and is hydrophilic due to Al–OH bond in its internal structure, and various properties are obtained in a single material due to its multiple structure. HNT has been found to increase the hydrophilic property of the materials where it is embedded as additives.^71,72^ Literature studies indicated that the nanoclay additives prevented the water vapor transmission because they cause a rougher surface formation which also causes an increase in the surface contact area. In case of rough and hydrophilic surfaces, water penetrates between the protrusions and the hydrophilic character of the surface increases.^73^ Therefore, water vapor is adsorbed within the HNT-embedded fiber structure, and this results in lower water vapor permeability. It can be said that the entrapment of water vapor between the protrusions of the HNT-embedded nanofiber surface reduced the water vapor permeability and/or transmission. The tortuous pathways of surfaces including HNTs act as a barrier to water vapor and reduce the water vapor transmission.^74,75^ Besides the effect of HNTs, the composite nanofibers including the electrosprayed photocatalysts, both TiO_2_ and ZnO, slightly reduced the water vapor transmission of nanofibers. This result is due to the high water vapor absorption capacity of TiO_2_^76^ and hydrophilic structure of ZnO.^77^ Similar findings were also reported in the literature by Hashmi et al. (2019b), and they fabricated the PAN/CuO composite nanofiber for antibacterial applications for respiratory mask applications. It was found that the values of water uptake (this term is opposite of WVP) for neat PAN, 0.25% CuO, 0.50% CuO, 0.75% CuO, and 1.00% CuO were 4729, 5163, 5708, 6497, and 6839 g/m^2^/day, respectively.^22^ In their study, the reduction of WVTR was explained with the hydrophilic properties of CuO.

### 3.3 Filtration Efficiency Test Results

**Figure 7:**
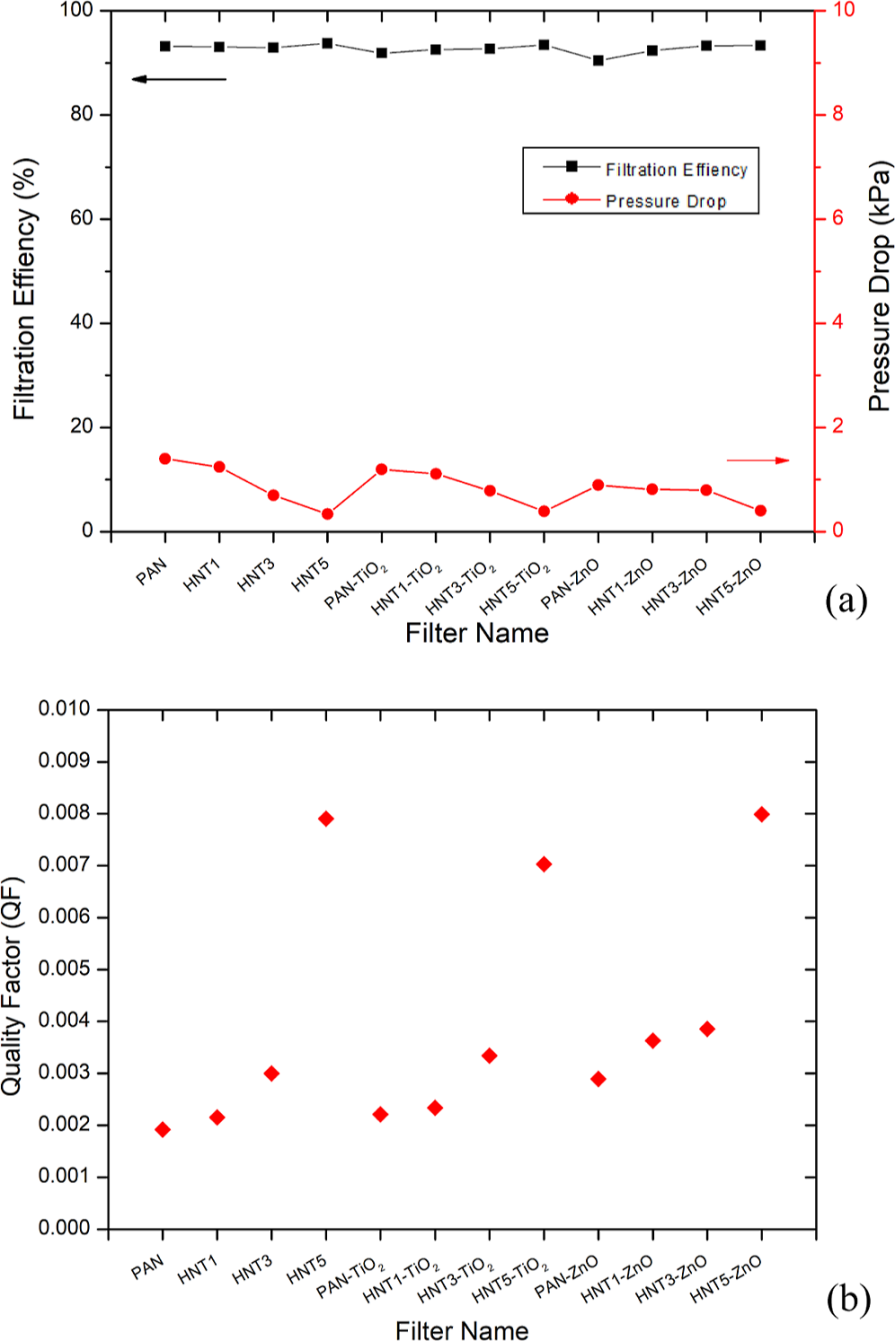
Filter efficiency and pressure drop values (a) and QF values of bare and PAN composite nanofiber filters (b).

Figure 7a shows the filter efficiency and pressure drop values of bare and composite nanofiber filters. The experiments were performed with 0.3 μm NaCl aerosol at a flow rate of 95 L/min. It was observed that with the increase in the concentration of HNTs in nanofiber composite filters from 1 to 5 wt %, the filtration efficiency of PAN/HNT composite nanofiber filters also slightly increased. Considering the general filtration efficiencies of the filters, the filtration efficiencies of PAN/HNT composite nanofibers were measured to be 93.2, 93.1, 93.2, and 93.1% at HNT concentrations of 0, 1, 3, and 5 wt %, respectively, and the fiber diameters of PAN/HNT composite nanofibers at HNT concentrations of 0, 1, and 3 wt % did not increase significantly. The tubular structure of HNT provides a hierarchical structure that could be more beneficial for PM removal efficiency, and this hierarchical structure creates an adsorbent surface for PM. The possibility of controlled high surface area in the HNT structure may help to capture smaller particles.^78^

Pressure drop values are generally measured below 1 kPa. The increase in the HNT content caused a decrease in pressure drop values in all filters, while the filter efficiency values were kept constant. The pressure drop values of PAN/HNT composite nanofibers were measured to be 1399, 1240, 898, and 337 kPa at HNT concentrations of 0, 1, 3, and 5 wt %, respectively. The low pressure drop value is an important parameter as the filters also reduce the energy demand. In this study, the reduction of pressure values by adding HNTs is shown to be beneficial in real air filtration applications. The addition of TiO_2_ and ZnO to the filter structure did not cause any serious change in both pressure drop and filter efficiency values of the nanofiber filters. Deng et al. (2022) reported that curved riboon PVA nanofiber membranes showed 99% removal for PM1.0 to be used in face filters, while the pressure drop was 57.5 Pa.^79^

Figure 7b shows the quality factor (QF) values of nanofibers. High filter efficiency, low pressure drop values, and higher concentrations of HNTs increase the QF values of nanofibers. The nanofiber coded as HNT5 showed the highest QF value of 0.08. HNT5 had the lowest pressure drop value and high filtration efficiency. Chen et al. (2019) reported a high QF value of 0.039 in P25-beads/PAN filters in their filtration test with multiple aerosol particles of 30–500 nm.^25^ Deng et al. (2023) reported a high QF value of 0.2 in the convex structure in the surface of thermal-crosslinked sodium phytate/PVA (T-PANa/PVA) fiber filters in their filtration test with PM 2.5.^80^ Bansal and Purwar (2021) fabricated PAN composite filters with zinc oxide and modified montmorillonite. It was observed that with the increasing ZnO–Mt ratio in the fabricated nanofibers, the value of QF increased initially and then a decrease was found. While the QF value in bare PAN filters was 0.0012, it was recorded as 0.0014, 0.0082, 0.0259, and 0.0009 for 0.25%, 0.5, 0.75, and 1 ZnO–Mt ratios, respectively.^21^

### 3.4 Antibacterial Activity of Nanofibers

The antibacterial performance of nanofibers were initially evaluated with agar disc diffusion test. From test results, a zone of inhibition was not observed around the nanofiber composite filters. However, there were no growth observed under the filter discs, which indicates that the composite filters have stationary non-releasing antibacterial agents on the surface. Similarly, Bansal and Purwar (2021) have found no zone of inhibition around (PAN)/ZnO-modified montmorillonite nanofiber mats while no bacterial growth was detected beneath the nanofiber, suggesting that the nanofiber mats have an antibacterial effect through infiltration or barrier mechanism. The bacterial cell in direct contact with the fibrous matrix was destructed since the ZnO nanoparticles were not released from the polymeric matrix into the agar medium due to weak bond between acrylonitrile and Zn cations.^21^

**Figure 8:**
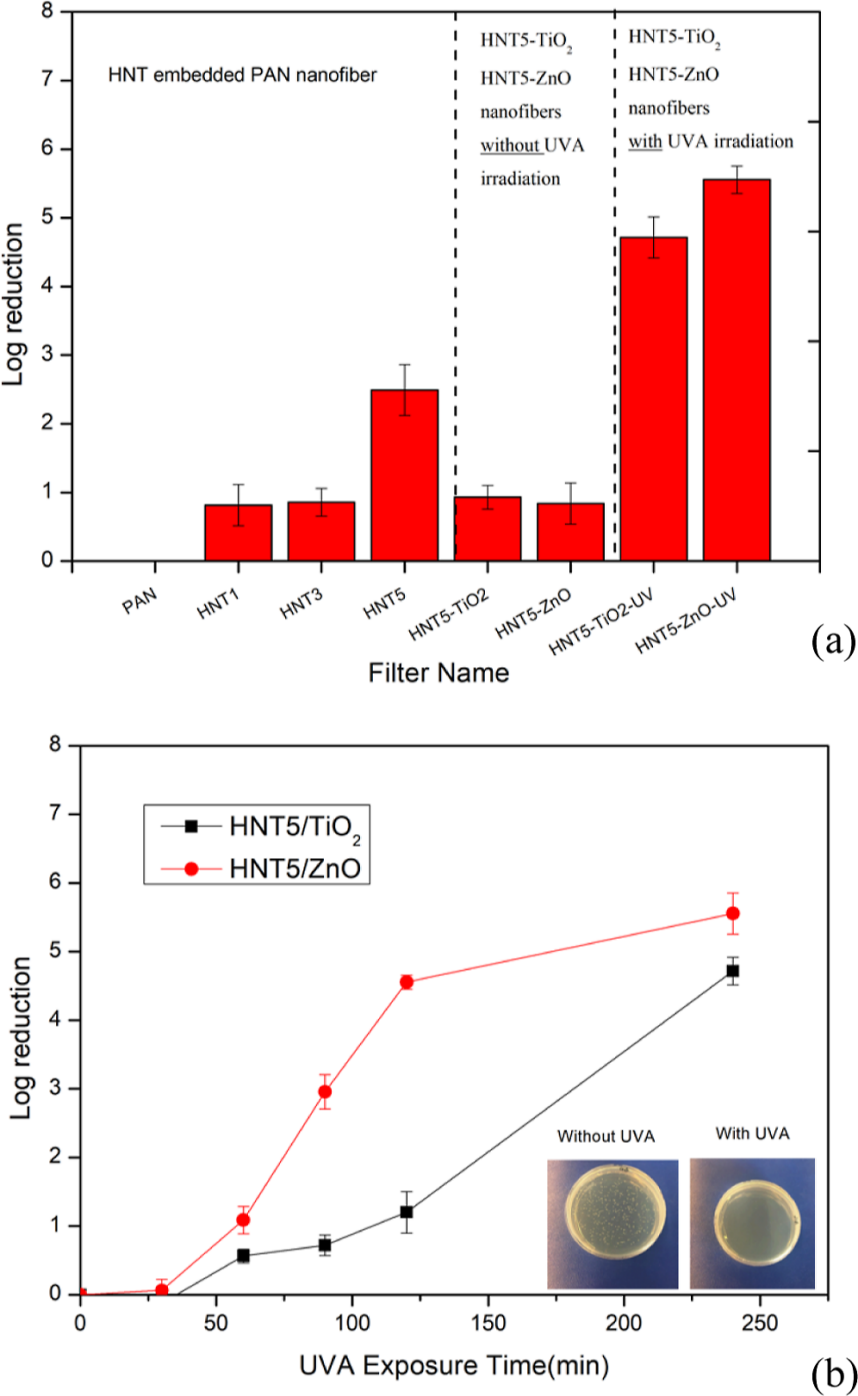
Antibacterial activity plots (a) log reduction values of all nanofibers, (b) time change of log reduction of HNT5-TiO_2_ and HNT5-ZnO nanofibers under UVA exposure (low inset: Petri images of the ZnO filter with UVA and without UVA. Note: photo was taken by one of the authors).

Antibacterial effectiveness were further evaluated by plate counting method for bare, HNT, HNT/TiO_2_, and HNT/ZnO additive filters as shown in Figure 8a. Antibacterial activity (% reduction and log reduction) of nanofibers was calculated using eqs 4 and 5 after 240 min of contact time (Figure 8). The PAN nanofiber was used as a control sample and showed no antibacterial activity. Considering the effect of different HNT ratios on antibacterial activity, the HNT5 nanofiber showed the highest bacterial reduction of 99.68%. Due to its high antibacterial activity and better filtration performance, the HNT5 nanofiber was used as a base material for the fabrication of TiO_2_ and ZnO nanofibers. The antibacterial activity of HNT5-TiO_2_ and HNT5-ZnO-sprayed nanofibers decreased to 88.27 and 85.56%, respectively, without UVA exposure. These results may cause from the decrease of the active surface area of HNT after TiO_2_ and ZnO nanoparticle incorporation. On the other hand, the antibacterial activity efficiency of the HNT5-TiO_2_ and HNT5-ZnO nanofibers was recovered and further increased over 99.99 under photocatalytic activity in the presence of UVA irradiation. The log reduction of HNT5-TiO_2_-sprayed nanofibers was found as 0.93 without UVA irradiation, while it was increased to 4.71 after UVA irradiation, which was a very effective 5-fold increase. For HNT5-ZnO-sprayed nanofibers, the antibacterial activity in the absence and presence of UVA exposure was determined as 0.84-log reduction and 5.56-log reduction, respectively. The comparison of the log reduction showed an increase of 6.6 times with a photocatalytic activity of ZnO.

In Figure 8b, HNT5-TiO_2_ and HNT5-ZnO composite nanofibers were compared in terms of time-dependent *versus* log-reduction values that gave two successful results upon UVA exposure. For both samples, the antibacterial activity efficiencies were gradually increased with an increasing UVA exposure time. The highest antibacterial effect was observed for the HNT5-ZnO sample with UVA irradiation, and a 5.56-log reduction was obtained. Correspondingly, Chen et al. (2019) in their study on the fabrication of facial masks prepared composite nanofibers with the addition of TiO_2_, which exhibited excellent photocatalytic activity for methylene blue and strong antibacterial activity. The fabricated composite nanofiber filters performed nine times better under UVA light than the filter PAN.^81^

**Figure 9:**
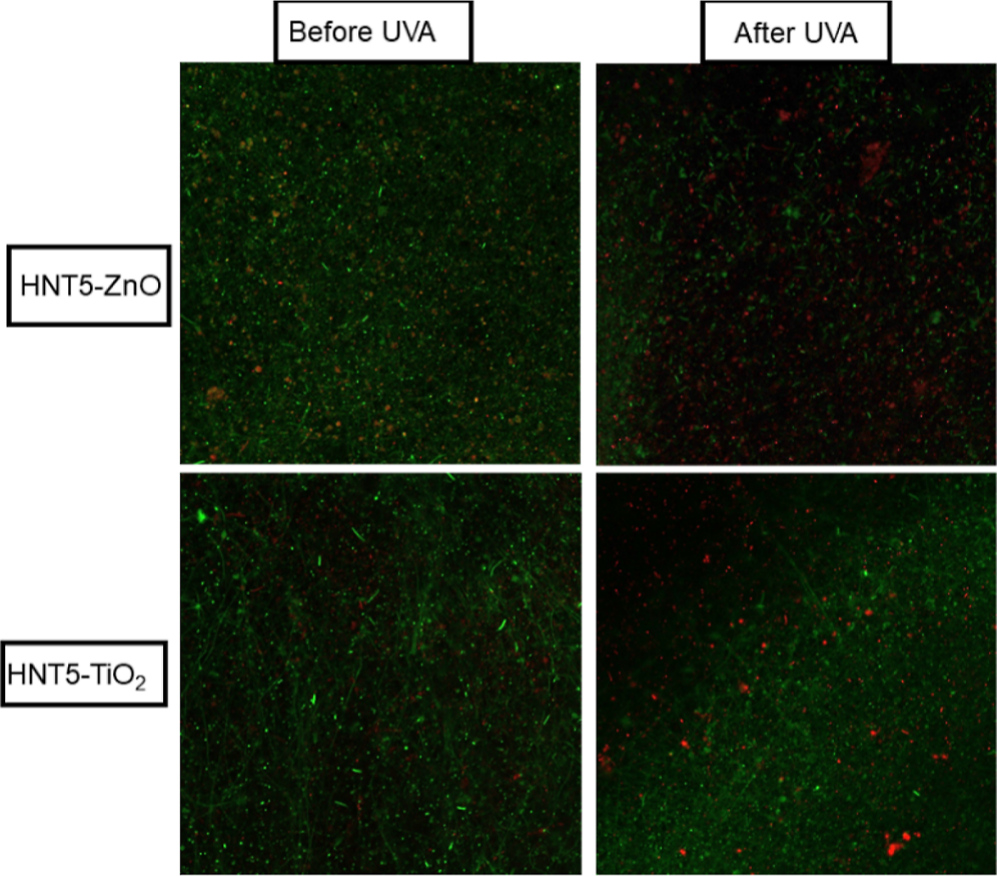
CLSM Analysis images of (a) HNT5-TiO_2_ and (b) HNT5-ZnO.

Nigussie et al., (2018) investigated antibacterial effect of Ag–TiO_2_ and Ag–ZnO nanoparticles against *E. coli* and found that the antibacterial properties of Ag–ZnO nanomaterials were more effective. Since ZnO has a higher electron mobility than TiO_2_, this accelerates electron transfer.^82^ The position of the valence band of ZnO is lower than that of TiO_2_, so the oxidation potential of the hydroxyl radical produced by ZnO is higher than that of the hydroxyl radical produced by TiO_2_.^83^ Three mechanisms have been described for the antibacterial activity of ZnO.^84^ The destruction of the cell wall of ZnO takes place when it comes into contact with bacteria, the release of Zn^2+^ ions, and the formation of reactive oxygen species (Bansal and Purwar, 2021). The contact between metal oxides and bacterial cells leads to oxidation and the formation of reactive oxygen groups such as O^2–^, −OH, and H_2_O_2_. These free radicals damage the cell walls of the bacteria altering the integrity and permeability of the membranes, leading to the death of the bacterial cells.^85−87^Figure 9 shows the CLSM images of two filters (HNT5/TiO_2_ and HNT5/ZnO) before and after UVA exposure, which is used to evaluate the antibacterial properties of them. The aim of this analysis is to detect both live and dead bacteria by using the photocatalytic effect and to show the antibacterial properties of the filters. It was observed that the green colors, which are an indicator of vitality, were predominant in the nanofiber filters that were not exposed to UVA and red colors representing dead bacteria increased with the photocatalytic effect after 1 h of UVA exposure. The formation of antibacterial activities usually occurs through the interaction of negatively charged bacterial cells and positively charged metal oxide nanoparticles.^88^ Goei and Lim (2014) used silver-modified mesoporous TiO_2_ materials and reported that the intensity of red fluorescence in CLSM images gradually increased after 30 min and 1 h of UV exposure against *E. coli* bacteria.^89^ ZnO and TiO_2_ nanoparticles can kill bacteria mainly by the ROS (reactive oxygen species) mechanism in the presence of UVA light.

## 4 Conclusions

The need for the development of innovative filtration materials and technologies for improving indoor air quality has significantly increased due to the increased time spent indoors since the beginning of COVID-19 pandemic. Particular attention should be paid to ensure that the materials to be used are easy to obtain, cheap, less energy consuming, less harmful to the environment, and antibacterial. The novelty of this study is the use of electrospraying and electrospinning methods together to prepare HNT-embedded antibacterial nanofibers to be used as filter materials and use HNTs as airborne filtration materials for the first time in the literature. Some novel aspects are also pointed out in the development of composite nanofilters for facial mask and/or indoor air filter applications such as less material consumption due to electrospinning process, ease of availability of HNTs, and the antibacterial effect of both TiO_2_ and ZnO nanoparticles. PAN/HNT/TiO_2_ and PAN/HNT/ZnO nanofiber filters were successfully fabricated by electrospinning method with various HNT ratios (1, 3, and 5 wt %) and integration of photocatalysts (TiO_2_ and ZnO) via electrospraying. The fabricated composite nanofiber filters have shown results that can be applied on a real scale. The effect of embedding HNTs and spraying photocatalysts enables us to fabricate nanofiber composite filters with lower pressure drop, high filtration efficiency, improved mechanical strength, and high antibacterial properties against *E. coli* and make nanofibers suitable and promising for facial mask and indoor air filter materials. Increasing the amount of HNT embedded into the PAN polymer also increased the air permeability values of the filters. HNT is also found to be a promising nano additive as to be used in face masks and air filter materials due to its cause in the reduction of the water vapor permeability values of the nanofibers. The addition of HNTs to nanofibers resulted in a dramatic increase in QF values, which is also a very important parameter for face masks. When the results were investigated from the point of antibacterial behavior of the filters, the effect of HNTs on antibacterial properties was very significant. The antibacterial effect of the filter was increased by the addition of 5% HNT to the polymer and contributed to 2.49 log reduction in bacterial colony formation. The integration of TiO_2_ and ZnO as catalysts into HNT-doped nanofiber filters significantly enhanced the antibacterial activity in the presence of UVA irradiation. HNT5-ZnO composite filter showed highest antibacterial activity with a 5.56-log reduction under UVA exposure.
